# Supplementary material for: Drug repurposing for aging research using model organisms
Source: Aging Cell. 2017 Jun 16;16(5):1006–15. doi: 10.1111/acel.12626 (PMC5595691; doi:10.1111/acel.12626)
Supplement: Supplementary file 7 — Data S1 Zip‐Archive of all report cards. [file ACEL-16-1006-s007.zip › RC_IXM.pdf]

IXM

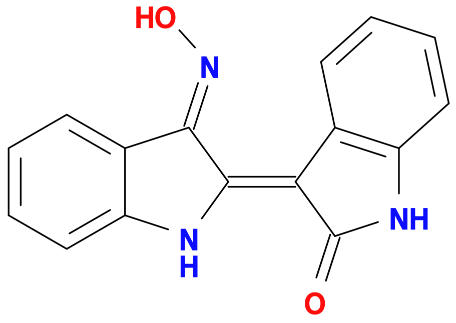

Database identifiers

ChEMBLCompound	CHEMBL216543
DrugBank	DB02052
eMolecules	1934826

Ranking

	Rank	Score
Drosophila	73/697	0.806
C. elegans	17/591	0.554

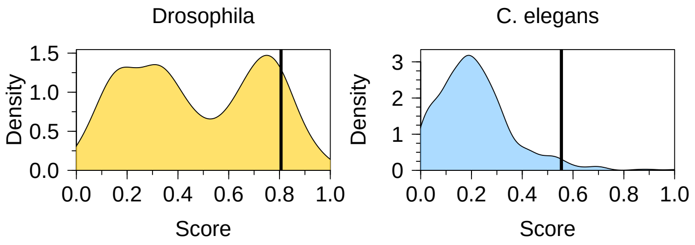

	Ageing implication	Domain conservation	Binding site conservation	Binding affinity	Bioavailability	Lipinski	Promiscuity	Purchasability	Drug approval	**Total**
Drosophila	1.0	0.973	0.907	0.796	(0.9)	0.0	-0.001	0.1	0.075	0.806
C. elegans	1.0	0.97	0.965	0.796	0.51	0.0	-0.001	0.1	0.075	0.554

Names

- (z)-1h,1'h-2,3'biindolylidene-3,2'-dione-3-oxime
- 3-3-(hydroxyamino)-1H-indol-2-yl-2-indolone
- BCBcMAP01$_0$00150$BiomolKI_0$00070
- BSPBio$_0$01108$I0404_sigma$
- Indirubin-38242;-oxime
- Indirubin-3prime;-monoxime
- Indirubin-3'-monoxime
- KBioGR$_0$00448$Lopac-I-0404$
- Lopac0$_0$00619$nchembio790-comp29$
- Tocris-1813

Roles

ChEBI entry None has no roles

Status

Approved drug (according to ChEMBL)	No
Classification (according to DrugBank)	experimental
Number of Rule of 5 violations	0
Binding affinity to original target in log units (RF-Score prediction)	6.36
Burns *C. elegans* bioavailability prediction	2.98

Compound Target Characteristics

Glycogen synthase kinase-3 beta

Best gene implication in ageing for this target family came from gene P49841 via mapping the annotation from Ensembl ENSG00000082701 via mapping the annotation from EntrezGene 2932 via mapping the annotation from GenAgeHuman 0097 annotated in GenAge release 17.

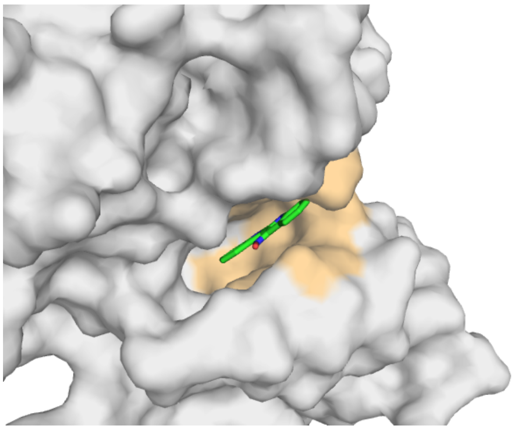

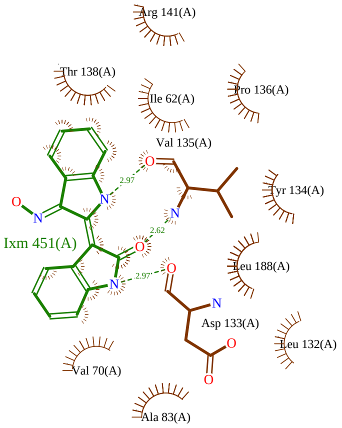

protein	amino acids contacts (binding site)
PDB:1q41:chainA:P49841	I V A L D Y V P T R L
tr:Q6FI27:Q6FI27_HUMAN	I V A L D Y V P T R L
sp:P49841:GSK3B_HUMAN	I V A L D Y V P T R L
sp:P18266:GSK3B_RAT	I V A L D Y V P T R L
tr:Q5KU03:Q5KU03_MOUSE	I V A L D Y V P T R L
sp:Q9WV60:GSK3B_MOUSE	I V A L D Y V P T R L
tr:E9QAQ5:E9QAQ5_MOUSE	I V A L D Y V P T R L
tr:B6IDN4:B6IDN4_DROME	I V A L E Y I P T K L
sp:P18431:SGG_DROME	I V A L E Y I P T K L
tr:A4V3W2:A4V3W2_DROME	I V A L E Y I P T K L
tr:A4V3W1:A4V3W1_DROME	I V A L E Y I P T K L
tr:Q0KHW6:Q0KHW6_DROME	I V A L E Y I P T K L
tr:E6PBX9:E6PBX9_DROME	I V A L E Y I P T K L
tr:H5V852:H5V852_DROME	I V A L E Y I P T K L
sp:Q9U2Q9:GSK3_CAEEL	I V A L E Y V P T R L
sp:P50873:MRK1_YEAST	V V A L D Y M P S Q L

protein	whole protein ident	simil	domain-based ident	simil	contact-based ident	simil
PDB:1q41:chainA:P49841	1.0	1.0	1.0	1.0	1.0	1.0
tr:Q6FI27:Q6FI27_HUMAN	1.0	1.0	1.0	1.0	1.0	1.0
sp:P49841:GSK3B_HUMAN	1.0	1.0	1.0	1.0	1.0	1.0
sp:P18266:GSK3B_RAT	0.99	1.0	1.0	1.0	1.0	1.0
tr:Q5KU03:Q5KU03_MOUSE	0.99	1.0	1.0	1.0	1.0	1.0
sp:Q9WV60:GSK3B_MOUSE	0.99	1.0	1.0	1.0	1.0	1.0
tr:E9QAQ5:E9QAQ5_MOUSE	0.96	0.97	0.96	0.96	1.0	1.0
tr:B6IDN4:B6IDN4_DROME	0.58	0.71	0.85	0.96	0.73	0.91
sp:P18431:SGG_DROME	0.59	0.74	0.85	0.96	0.73	0.91
tr:A4V3W2:A4V3W2_DROME	0.59	0.74	0.85	0.96	0.73	0.91
tr:A4V3W1:A4V3W1_DROME	0.52	0.66	0.85	0.96	0.73	0.91
tr:Q0KHW6:Q0KHW6_DROME	0.49	0.63	0.85	0.96	0.73	0.91
tr:E6PBX9:E6PBX9_DROME	0.18	0.26	0.52	0.65	0.73	0.91
tr:H5V852:H5V852_DROME	0.28	0.35	0.85	0.96	0.73	0.91
sp:Q9U2Q9:GSK3_CAEEL	0.61	0.76	0.81	0.95	0.91	0.96
sp:P50873:MRK1_YEAST	0.31	0.57	0.53	0.84	0.64	0.86

sgg (FBgn0003371) associated phenotypes

cell non-autonomous, cell polarity defective, chemical sensitive, circadian rhythm defective, developmental rate defective, germline clone, heat sensitive, lethal - all die before end of P-stage, lethal - all die before end of pupal stage, maternal effect, meiotic cell cycle defective, mitotic cell cycle defective, neuroanatomy defective, neurophysiology defective, non-rescuable maternal effect, planar polarity defective, rescuable maternal effect, short lived, smell perception defective, somatic clone, some die during pharate adult stage, some die during pupal stage, some die during third instar larval stage

(Information from FlyBase)

sgg (UniProt:P18431) annotation

Function: Required for several developmental events such as syncytial blastoderm formation and embryonic segmentation. Is involved in transcriptional regulation. Required for arm phosphorylation. Wg signaling operates by inactivating the sgg repression of en autoactivation. Negatively controls the neuromuscular junction (NMJ) growth in presynaptic motoneurons. Plays a role in the regulation of microtubule dynamics and actin cytoskeleton during embryogenesis. Required for phosphorylation of sra in activated eggs. Essential for completion of meiosis, possibly by triggering calcineurin activation via sra phosphorylation. Phosphorylates microtubule-associated protein futsch in axons. (PubMed:15269269, PubMed:16570248, PubMed:16949836, PubMed:2113617, PubMed:2118107, PubMed:22421435, PubMed:7529201, PubMed:8467811).
Subunit: Interacts with cos. (PubMed:1335365,PubMed:15691767).
Subcellular location: Cytoplasm. Nucleus. Cytoplasm, cytoskeleton, microtubule organizing center, centrosome. Cytoplasm, cell cortex. Cell junction, synapse. Cell projection, axon. Note=In syncytial embryos, detected at the centrosomes throughout the cell cycle, and in the mitotic spindle and pseudocleavage furrows invaginating from the cell cortex during mitosis. Concentrated at the growing end of membranes during the cellularization process. After cellularization, localized to the centrosomes during mitosis and to the nucleus at the end of telophase. Enriched in the presynaptic side of the neuromuscular junction, with some signal detected also in axonal branches.
Tissue specificity: Expressed in ovaries and activated eggs (at protein level). Expression is over all the embryo at all stages, no local accumulation is observed. (PubMed:2118107, PubMed:22421435, PubMed:8467811).
Developmental stage: Isoform SGG46 is expressed at low levels in 12-24 hours embryos. Isoform Zygotic and isoform SGG39 are expressed in 12-24 hours embryos and present throughout the larval, pupal and adult stages (at protein level). Isoform Zygotic is expressed maternally and zygotically but reduced throughout later embryonic development. Expression persists throughout larval stages. (PubMed:2118107, PubMed:8467811).
Disruption phenotype: Mutants display an overdeveloped neuromuscular junction (NMJ), with the number of boutons greatly increased. (PubMed:15269269).

(Information from UniProt)

gsk-3 (WBGene00001746) associated phenotypes

axon morphology variant, larval lethal, lethal, locomotion variant, sterile

(Information from WormBase)

gsk-3 (UniProt:Q9U2Q9) annotation

Function: Phosphorylates oma-1, a regulator of the oocyte-to- embryo transition, enabling its degradation. Phosphorylates skn-1, preventing it from accumulating in nuclei and thus inhibiting phase II gene expression in the oxidative stress defense. Involved in mesendoderm specification and mitotic spindle orientation in EMS blastomeres. Thought to be a branch point in these processes as proteins downstream are not required. Negatively regulates Wnt signaling in vulval precursor cells and acts as a Wnt-independent repressor of med-1 and med-2 in the C lineage inhibiting mesoderm development. Required for normal lifespan and LiCl-induced lifespan extension. (PubMed:10444600, PubMed:11463373, PubMed:15572126, PubMed:16251270, PubMed:16289132, PubMed:16343905, PubMed:16930586, PubMed:17959600).

Subunit: Monomer (By similarity). Interacts with axl-1. (UniProtKB:Q9WV60, PubMed:17601533).

Disruption phenotype: Worms exhibit defects in endoderm specification and mitotic spindle alignment. Mutants show reduced degradation of oma-1 and have shortened lifespan. (PubMed:10444600).

(Information from UniProt)

Cyclin-dependent-like kinase 5

Best gene implication in ageing for this target family came from gene P48609 annotated in UniProt release 2014_02. Annotation GO 8340 (determination of adult lifespan) was Inferred from Mutant Phenotype

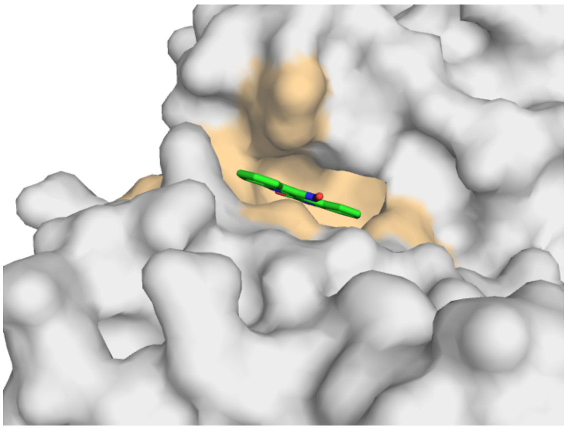

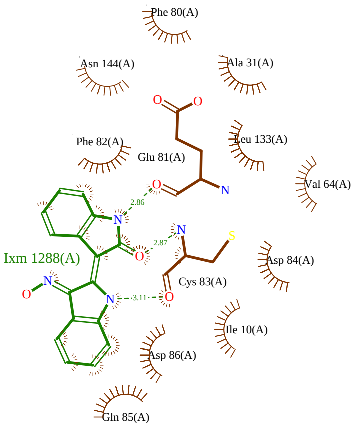

protein	amino acids contacts (binding site)											
PDB:1unh:chainA:Q00535	I	A	V	F	E	F	C	D	Q	D	L	N
sp:Q00535:CDK5_HUMAN	I	A	V	F	E	F	C	D	Q	D	L	D
sp:Q03114:CDK5_RAT	I	A	V	F	E	F	C	D	Q	D	L	D
sp:P49615:CDK5_MOUSE	I	A	V	F	E	F	C	D	Q	D	L	D
tr:Q543F6:Q543F6_MOUSE	I	A	V	F	E	F	C	D	Q	D	L	D
sp:P48609:CDK5_DROME	I	A	V	F	E	H	C	D	Q	D	L	D
tr:G5ECH7:G5ECH7_CAEEL	I	A	V	F	E	Y	C	D	Q	D	L	D
sp:P17157:PHO85_YEAST	L	A	V	F	E	F	M	D	N	D	L	D

protein	whole protein ident	simil	domain-based ident	simil	contact-based ident	simil
PDB:1unh:chainA:Q00535	1.0	1.0	1.0	1.0	1.0	1.0
sp:Q00535:CDK5_HUMAN	1.0	1.0	1.0	1.0	0.92	0.98
sp:Q03114:CDK5_RAT	0.99	1.0	0.99	1.0	0.92	0.98
sp:P49615:CDK5_MOUSE	1.0	1.0	1.0	1.0	0.92	0.98
tr:Q543F6:Q543F6_MOUSE	1.0	1.0	1.0	1.0	0.92	0.98
sp:P48609:CDK5_DROME	0.78	0.93	0.79	0.94	0.83	0.9
tr:G5ECH7:G5ECH7_CAEEL	0.74	0.93	0.75	0.93	0.83	0.97
sp:P17157:PHO85_YEAST	0.54	0.82	0.56	0.83	0.67	0.79

Cdk5 (FBgn0013762) associated phenotypes
neuroanatomy defective, short lived

(Information from FlyBase)

Cdk5 (UniProt:P48609) annotation
Function: Probably involved in the control of the cell cycle. Interacts with D1 and D3-type G1 cyclins. Possible regulator of neuronal differentiation and/or development (By similarity).
Tissue specificity: Abundantly expressed in all adult tissues. Lower levels found in larvae and early embryos. Barely detectable in late embryos.

(Information from UniProt)

cdk-5 (WBGene00000407) associated phenotypes
aldicarb resistant

(Information from WormBase)

cdk-5 (UniProt:G5ECH7) annotation
Function: Proline-directed serine/threonine-protein kinase which, in several motor neurons, promotes the polarized trafficking of synaptic vesicles and dense-core vesicles (DCV). In the ventral nerve cord, phosphorylates lin-10 and thereby prevents lin-10- mediated anterograde trafficking of the glutamate receptor glr-1 (PubMed:17671168, PubMed:21609829). Involved in the inhibition of glr-1 trafficking in hypoxic conditions (PubMed:22252129). In DA motor neurons but not in DB motor neurons, regulates axonal transport of synaptic vesicle precursors by inhibiting dynein-mediated retrograde transport (PubMed:20510931). Regulates the trafficking of dense-core vesicles in DA and DB motor neurons by promoting anterograde trafficking to the axon and preventing dynein-dependent trafficking to the dendrite (PubMed:22699897). May regulate these processes in association with cdka-1/p35 (PubMed:17671168, PubMed:20510931). Activity may be regulated by cyy-1 (PubMed:20510931). Involved in synapse formation during DD motor neuron remodeling by regulating transport of disassembled synaptic material to the new synaptic sites probably by activating the motor protein unc-104/kinesin-3 (PubMed:21609829). Regulates microtubule polarity in the dendrite of DB motor neurons (PubMed:22699897). (PubMed:17671168, PubMed:20510931, PubMed:21609829, PubMed:22252129, PubMed:22699897).
Cofactor: Mg(2+) Evidence=(PubMed:20510931); Note=Binds 2 Mg(2+) ions. (UniProtKB:P24941);
Subunit: Heterodimer composed of a catalytic subunit cdk-5 and a regulatory subunit cdka-1. Interaction with cdka-1 is required for cdk-5 activation. (PubMed:22699897).
Subcellular location: Cytoplasm (PubMed:20510931). Cell projection, dendrite (PubMed:20510931). Note=Localizes predominantly to presynaptic sites and in dendrites as faint puncta. (PubMed:20510931).
Disruption phenotype: Several glr-1-dependent behaviors are affected including an absence of backward locomotion after nose- touching stimuli and a reduction in reverse locomotion (PubMed:17671168). In L4 mutants, incomplete elimination of ventral rab-3-positive synaptic vesicles associated with a delay in the formation of dorsal rab-3-positive synaptic vesicles in DD motor neurons. Normal formation of ventral synapses in DD motor neurons at the L1 stage (PubMed:21609829). In addition, mutants have an increase in anterograde dense-core vesicle trafficking and in the number of plus-end-out microtubules in DB motor neuron dendrites (PubMed:22699897). Reduced sensitivity to the acetylcholine esterase inhibitor aldicarb (PubMed:22699897). (PubMed:17671168,

PubMed:21609829, PubMed:22699897).

(Information from UniProt)
